# 2,3-*O*-Isopropyl­idene-1-*O*-*p*-tolyl­sulfonylglycerol

**DOI:** 10.1107/S1600536809015037

**Published:** 2009-05-07

**Authors:** Piotr Kuś, Marcin Rojkiewicz, Grzegorz Zięba, Peter G. Jones

**Affiliations:** a Department of Chemistry, University of Silesia, 9 Szkolna Street, 40-006 Katowice, Poland; b Institut für Anorganische und Analytische Chemie, Technische Universität Braunschweig, Postfach 3329, 38023 Braunschweig, Germany

## Abstract

In the title compound, C_13_H_18_O_5_S, the five-membered ring has an envelope conformation. The packing involves four C—H⋯O inter­actions, three of which combine to form layers of mol­ecules parallel to the *bc* plane.

## Related literature

For related literature, see: Baer & Fischer (1948 ▶); Jones *et al.* (2003 ▶); Kazemi *et al.* (2007 ▶); Ouchi *et al.* (1990 ▶). The structure of a related derivative is presented in the following paper, see: Kuś *et al.* (2009 ▶).
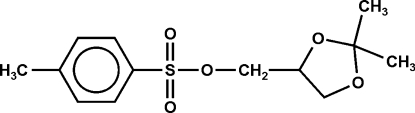


## Experimental

### 

#### Crystal data

C_13_H_18_O_5_S
*M* *_r_* = 286.33
Monoclinic, 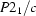

*a* = 15.143 (2) Å
*b* = 5.7297 (9) Å
*c* = 15.665 (2) Å
β = 90.385 (3)°
*V* = 1359.2 (4) Å^3^
*Z* = 4
Mo *K*α radiation
μ = 0.25 mm^−1^
*T* = 133 K
0.40 × 0.20 × 0.05 mm

#### Data collection

Bruker SMART 1000 CCD area-detector diffractometer
Absorption correction: none
12509 measured reflections
3357 independent reflections
2339 reflections with *I* > 2σ(*I*)
*R*_int_ = 0.111

#### Refinement

*R*[*F* ^2^ > 2σ(*F* ^2^)] = 0.051
*wR*(*F* ^2^) = 0.114
*S* = 1.05
3357 reflections
175 parameters
H-atom parameters constrained
Δρ_max_ = 0.31 e Å^−3^
Δρ_min_ = −0.48 e Å^−3^

### 

Data collection: *SMART* (Bruker, 1998 ▶); cell refinement: *SAINT* (Bruker, 1998 ▶); data reduction: *SAINT*; program(s) used to solve structure: *SHELXS97* (Sheldrick, 2008 ▶); program(s) used to refine structure: *SHELXL97* (Sheldrick, 2008 ▶); molecular graphics: *XP* (Siemens, 1994 ▶); software used to prepare material for publication: *SHELXL97*.

## Supplementary Material

Crystal structure: contains datablocks I, global. DOI: 10.1107/S1600536809015037/bt2935sup1.cif

Structure factors: contains datablocks I. DOI: 10.1107/S1600536809015037/bt2935Isup2.hkl

Additional supplementary materials:  crystallographic information; 3D view; checkCIF report

## Figures and Tables

**Table 1:** Hydrogen-bond geometry (Å, °)

*D*—H⋯*A*	*D*—H	H⋯*A*	*D*⋯*A*	*D*—H⋯*A*
C16—H16⋯O1^i^	0.95	2.60	3.212 (2)	122
C17—H17*C*⋯O1^ii^	0.98	2.65	3.369 (3)	131
C17—H17*B*⋯O1^iii^	0.98	2.66	3.558 (3)	153
C5—H5*A*⋯O2^iv^	0.98	2.64	3.509 (3)	148

Symmetry codes: (i) 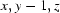; (ii) 
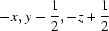
; (iii) 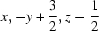; (iv) 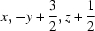.

**Fig. 1.**
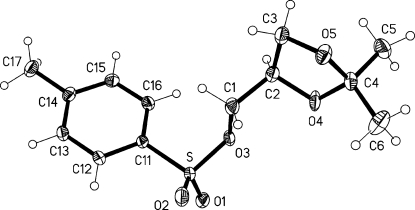
The title compound in the crystal structure. Displacement ellipsoids represent 50% probability levels.

**Fig. 2.**
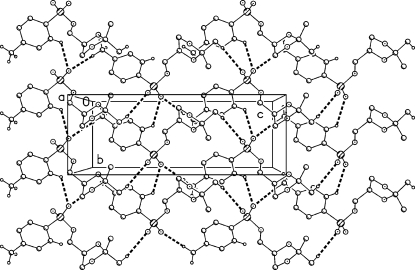
Packing diagram of the title compound in the region x≈ 1/4. H atoms not involved in H bonding (thick dashed lines) are omitted for clarity.

## supplementary crystallographic information

### Comment

Isopropylidene and tosyl groups are often used as protecting or activating units in polyhydroxyalkyl compounds used for synthesis of sugar-like derivatives. Recently we described the crystal structure of *L*-arabitol tosylate protected by two isopropylidene groups (Jones *et al.*, 2003). *D*,*L*-Isopropylideneglycerol is an important substrate for the synthesis of many derivatives of glycerol; the isopropylidene protecting group is a convenient form of protection for the two vicinal hydroxyls in the molecule of glycerol. After the reaction at the third, free hydroxyl group, acid hydrolysis of the protecting group leads to 1-*O*-substituted glycerol derivatives. In our experiments we used commercial (Aldrich) solketal as precursor for the protecting group. Tosylation of this compound leads to compound 1 (Baer & Fischer, 1948; Ouchi *et al.* 1990; Kazemi *et al.* 2007).

The molecule is shown in Fig. 1. Bond lengths and angles may be regarded as normal. The five-membered ring displays an envelope conformation, with approximate local mirror symmetry about O5 and the midpoint of C2—O4. The chain of five atoms from S to C3 displays an extended conformation, with torsion angles close to ±180°.

There are four weak C—H···O interactions with H···O between 2.6 and 2.7 Å. Three of these combine to form layers of molecules parallel to the *bc* plane at *x*≈ 1/4 (Fig. 2) and 3/4.

The structure of a related derivative is presented in the following paper (Kuś *et al.*, 2009).

### Experimental

The compound 1 was obtained according to method described by Kazemi *et al.* (2007). The analytical and spectroscopic data are consistent with the literature. Single crystals suitable for X-ray analysis were obtained by slow evaporation from petroleum ether.

NMR data: ^1^H NMR (CDCl_3_, 400 MHz): δ 7.80 (d, 2H), 7.35 (d, 2H), 4.28 (q, 1H), 4.06–3.95 (m, 3H), 3.78–3.75 (dd, 1H), 2.45 (s, 3H), 1.34 (s, 3H), 1.31 (s, 3H). - ^13^C NMR (100 MHz): δ 145.21, 132.81, 130.06, 128.15, 110.20, 73.05, 69.62, 66.35, 26.77, 25.28, 21.80.

IR data: —S(O_2_)—O— vibrations at 1177 (*versus*) and 1348 cm^-1^ (*s*); 1,3-dioxalone ring at 971 cm^-1^. There are no bands above 2990 cm^-1^.

### Refinement

Methyl H atoms were identified in difference syntheses and refined as idealized rigid groups (C—H 0.98 Å, H—C—H 109.5°) allowed to rotate but not tip. Other H atoms were included at calculated positions and refined using a riding model, with fixed C—H bond lengths of 0.95 Å (CH, aromatic), 0.99 Å (CH_2_) and 1.00 Å (CH, *sp*^3^); *U*_iso_(H) values were fixed at 1.2*U*_eq_ of the parent C atom (1.2*U*_eq_ for methyl H).

### Crystal data

**Table:**

C_13_H_18_O_5_S	*D*_x_ = 1.399 Mg m^−^^3^
*M**_r_* = 286.33	Melting point: 321 K
Monoclinic, *P*2_1_/*c*	Mo *K*α radiation, λ = 0.71073 Å
*a* = 15.143 (2) Å	Cell parameters from 5579 reflections
*b* = 5.7297 (9) Å	θ = 2.6–29.6°
*c* = 15.665 (2) Å	µ = 0.25 mm^−^^1^
β = 90.385 (3)°	*T* = 133 K
*V* = 1359.2 (4) Å^3^	Lath, colourless
*Z* = 4	0.40 × 0.20 × 0.05 mm
*F*(000) = 608	

### Data collection

**Table:**

Bruker SMART 1000 CCD area-detector diffractometer	2339 reflections with *I* > 2σ(*I*)
Radiation source: fine-focus sealed tube	*R*_int_ = 0.111
graphite	θ_max_ = 28.3°, θ_min_ = 1.3°
Detector resolution: 8.192 pixels mm^-1^	*h* = −19→20
ω scans	*k* = −7→7
12509 measured reflections	*l* = −20→20
3357 independent reflections	

### Refinement

**Table:**

Refinement on *F*^2^	Primary atom site location: structure-invariant direct methods
Least-squares matrix: full	Secondary atom site location: difference Fourier map
*R*[*F*^2^ > 2σ(*F*^2^)] = 0.051	Hydrogen site location: inferred from neighbouring sites
*wR*(*F*^2^) = 0.114	H-atom parameters constrained
*S* = 1.05	*w* = 1/[σ^2^(*F*_o_^2^) + (0.0462*P*)^2^ + 0.1472*P*] where *P* = (*F*_o_^2^ + 2*F*_c_^2^)/3
3357 reflections	(Δ/σ)_max_ = 0.001
175 parameters	Δρ_max_ = 0.31 e Å^−^^3^
0 restraints	Δρ_min_ = −0.47 e Å^−^^3^

### Special details

**Table:**

Geometry. All e.s.d.'s (except the e.s.d. in the dihedral angle between two l.s. planes) are estimated using the full covariance matrix. The cell e.s.d.'s are taken into account individually in the estimation of e.s.d.'s in distances, angles and torsion angles; correlations between e.s.d.'s in cell parameters are only used when they are defined by crystal symmetry. An approximate (isotropic) treatment of cell e.s.d.'s is used for estimating e.s.d.'s involving l.s. planes.
Refinement. Refinement of *F*^2^ against ALL reflections. The weighted *R*-factor *wR* and goodness of fit *S* are based on *F*^2^, conventional *R*-factors *R* are based on *F*, with *F* set to zero for negative *F*^2^. The threshold expression of *F*^2^ > σ(*F*^2^) is used only for calculating *R*-factors(gt) *etc*. and is not relevant to the choice of reflections for refinement. *R*-factors based on *F*^2^ are statistically about twice as large as those based on *F*, and *R*- factors based on ALL data will be even larger.

### Fractional atomic coordinates and isotropic or equivalent isotropic displacement parameters (Å^2^)

**Table:**

	*x*	*y*	*z*	*U*_iso_*/*U*_eq_	
S	0.20851 (4)	0.79423 (9)	0.35153 (3)	0.01847 (14)	
O1	0.14179 (11)	0.9403 (3)	0.38666 (8)	0.0271 (4)	
O2	0.28879 (11)	0.8986 (3)	0.32446 (9)	0.0287 (4)	
O3	0.22741 (10)	0.6118 (2)	0.42410 (8)	0.0194 (3)	
O4	0.31609 (11)	0.4304 (3)	0.56622 (8)	0.0278 (4)	
O5	0.44258 (10)	0.2270 (3)	0.54736 (8)	0.0243 (4)	
C1	0.30372 (15)	0.4563 (4)	0.41503 (12)	0.0229 (5)	
H1A	0.3592	0.5478	0.4141	0.027*	
H1B	0.2991	0.3661	0.3613	0.027*	
C2	0.30289 (15)	0.2953 (4)	0.49053 (11)	0.0210 (4)	
H2	0.2455	0.2093	0.4932	0.025*	
C3	0.38036 (15)	0.1234 (4)	0.49008 (13)	0.0243 (5)	
H3A	0.3618	−0.0325	0.5105	0.029*	
H3B	0.4053	0.1077	0.4321	0.029*	
C4	0.39249 (14)	0.3442 (4)	0.61038 (12)	0.0197 (5)	
C5	0.36304 (18)	0.1785 (4)	0.67969 (13)	0.0322 (6)	
H5A	0.3210	0.2579	0.7171	0.048*	
H5B	0.4145	0.1280	0.7132	0.048*	
H5C	0.3346	0.0420	0.6537	0.048*	
C6	0.44515 (18)	0.5465 (5)	0.64354 (15)	0.0371 (6)	
H6A	0.4604	0.6501	0.5961	0.056*	
H6B	0.4994	0.4889	0.6708	0.056*	
H6C	0.4102	0.6327	0.6854	0.056*	
C11	0.16470 (14)	0.6269 (3)	0.26750 (11)	0.0162 (4)	
C12	0.17492 (14)	0.7085 (4)	0.18417 (11)	0.0184 (4)	
H12	0.2062	0.8489	0.1731	0.022*	
C13	0.13833 (14)	0.5797 (4)	0.11806 (12)	0.0202 (5)	
H13	0.1436	0.6356	0.0612	0.024*	
C14	0.09418 (14)	0.3717 (4)	0.13239 (12)	0.0192 (4)	
C15	0.08507 (14)	0.2955 (4)	0.21663 (12)	0.0201 (4)	
H15	0.0547	0.1537	0.2277	0.024*	
C16	0.11935 (14)	0.4224 (4)	0.28417 (12)	0.0188 (4)	
H16	0.1119	0.3700	0.3412	0.023*	
C17	0.05788 (16)	0.2279 (4)	0.06012 (13)	0.0259 (5)	
H17A	0.0939	0.0872	0.0532	0.039*	
H17B	0.0591	0.3195	0.0073	0.039*	
H17C	−0.0031	0.1830	0.0727	0.039*	

### Atomic displacement parameters (Å^2^)

**Table:**

	*U*^11^	*U*^22^	*U*^33^	*U*^12^	*U*^13^	*U*^23^
S	0.0273 (3)	0.0169 (2)	0.0112 (2)	−0.0003 (2)	−0.00206 (17)	0.0006 (2)
O1	0.0413 (11)	0.0222 (8)	0.0180 (7)	0.0097 (7)	−0.0027 (7)	−0.0032 (6)
O2	0.0373 (10)	0.0323 (9)	0.0166 (7)	−0.0122 (8)	−0.0038 (6)	0.0014 (6)
O3	0.0256 (9)	0.0215 (7)	0.0111 (6)	0.0052 (6)	−0.0013 (5)	0.0010 (5)
O4	0.0350 (10)	0.0365 (9)	0.0117 (6)	0.0169 (7)	−0.0068 (6)	−0.0064 (6)
O5	0.0205 (8)	0.0315 (9)	0.0207 (7)	0.0047 (7)	−0.0018 (6)	−0.0069 (6)
C1	0.0273 (13)	0.0260 (11)	0.0154 (9)	0.0079 (10)	0.0013 (8)	−0.0016 (8)
C2	0.0252 (12)	0.0222 (10)	0.0157 (9)	0.0014 (10)	−0.0013 (8)	−0.0029 (8)
C3	0.0284 (13)	0.0259 (11)	0.0185 (10)	0.0041 (10)	−0.0061 (8)	−0.0038 (8)
C4	0.0181 (11)	0.0271 (12)	0.0141 (9)	0.0026 (9)	−0.0020 (7)	−0.0006 (8)
C5	0.0432 (15)	0.0325 (13)	0.0209 (10)	0.0022 (12)	0.0017 (10)	0.0071 (10)
C6	0.0373 (16)	0.0429 (15)	0.0310 (12)	−0.0098 (12)	0.0041 (10)	−0.0151 (11)
C11	0.0203 (11)	0.0149 (9)	0.0132 (8)	0.0030 (8)	−0.0019 (7)	−0.0002 (7)
C12	0.0233 (11)	0.0179 (9)	0.0141 (8)	−0.0003 (9)	0.0012 (8)	0.0030 (8)
C13	0.0256 (12)	0.0214 (11)	0.0135 (9)	0.0027 (9)	−0.0014 (8)	0.0016 (8)
C14	0.0186 (11)	0.0213 (10)	0.0178 (9)	0.0034 (9)	−0.0011 (8)	−0.0040 (8)
C15	0.0218 (11)	0.0174 (9)	0.0211 (9)	−0.0032 (9)	0.0003 (8)	−0.0003 (9)
C16	0.0216 (12)	0.0191 (10)	0.0156 (9)	0.0004 (9)	0.0022 (8)	0.0020 (8)
C17	0.0303 (13)	0.0271 (12)	0.0203 (10)	−0.0024 (10)	−0.0022 (9)	−0.0069 (9)

### Geometric parameters (Å, °)

**Table:**

S—O2	1.4220 (16)	C15—C16	1.382 (3)
S—O1	1.4253 (16)	C1—H1A	0.9900
S—O3	1.5694 (14)	C1—H1B	0.9900
S—C11	1.7552 (19)	C2—H2	1.0000
O3—C1	1.467 (2)	C3—H3A	0.9900
O4—C2	1.429 (2)	C3—H3B	0.9900
O4—C4	1.432 (2)	C5—H5A	0.9800
O5—C4	1.418 (2)	C5—H5B	0.9800
O5—C3	1.426 (2)	C5—H5C	0.9800
C1—C2	1.500 (3)	C6—H6A	0.9800
C2—C3	1.532 (3)	C6—H6B	0.9800
C4—C6	1.498 (3)	C6—H6C	0.9800
C4—C5	1.512 (3)	C12—H12	0.9500
C11—C16	1.384 (3)	C13—H13	0.9500
C11—C12	1.396 (2)	C15—H15	0.9500
C12—C13	1.384 (3)	C16—H16	0.9500
C13—C14	1.386 (3)	C17—H17A	0.9800
C14—C15	1.398 (3)	C17—H17B	0.9800
C14—C17	1.502 (3)	C17—H17C	0.9800
			
O2—S—O1	118.55 (10)	O4—C2—H2	110.4
O2—S—O3	110.12 (9)	C1—C2—H2	110.4
O1—S—O3	103.75 (8)	C3—C2—H2	110.4
O2—S—C11	109.01 (9)	O5—C3—H3A	111.1
O1—S—C11	110.18 (10)	C2—C3—H3A	111.1
O3—S—C11	104.21 (8)	O5—C3—H3B	111.1
C1—O3—S	118.31 (11)	C2—C3—H3B	111.1
C2—O4—C4	108.77 (15)	H3A—C3—H3B	109.0
C4—O5—C3	106.32 (16)	C4—C5—H5A	109.5
O3—C1—C2	106.63 (16)	C4—C5—H5B	109.5
O4—C2—C1	108.62 (17)	H5A—C5—H5B	109.5
O4—C2—C3	104.44 (16)	C4—C5—H5C	109.5
C1—C2—C3	112.42 (17)	H5A—C5—H5C	109.5
O5—C3—C2	103.40 (16)	H5B—C5—H5C	109.5
O5—C4—O4	105.15 (14)	C4—C6—H6A	109.5
O5—C4—C6	108.76 (18)	C4—C6—H6B	109.5
O4—C4—C6	109.08 (19)	H6A—C6—H6B	109.5
O5—C4—C5	111.38 (18)	C4—C6—H6C	109.5
O4—C4—C5	108.81 (19)	H6A—C6—H6C	109.5
C6—C4—C5	113.32 (18)	H6B—C6—H6C	109.5
C16—C11—C12	121.23 (18)	C13—C12—H12	120.8
C16—C11—S	120.43 (14)	C11—C12—H12	120.8
C12—C11—S	118.32 (16)	C12—C13—H13	119.1
C13—C12—C11	118.33 (19)	C14—C13—H13	119.1
C12—C13—C14	121.90 (18)	C16—C15—H15	119.3
C13—C14—C15	118.20 (18)	C14—C15—H15	119.3
C13—C14—C17	121.64 (18)	C15—C16—H16	120.5
C15—C14—C17	120.16 (19)	C11—C16—H16	120.5
C16—C15—C14	121.30 (19)	C14—C17—H17A	109.5
C15—C16—C11	119.02 (18)	C14—C17—H17B	109.5
O3—C1—H1A	110.4	H17A—C17—H17B	109.5
C2—C1—H1A	110.4	C14—C17—H17C	109.5
O3—C1—H1B	110.4	H17A—C17—H17C	109.5
C2—C1—H1B	110.4	H17B—C17—H17C	109.5
H1A—C1—H1B	108.6		
			
O2—S—O3—C1	−42.19 (16)	O2—S—C11—C16	145.72 (17)
O1—S—O3—C1	−170.05 (14)	O1—S—C11—C16	−82.58 (19)
C11—S—O3—C1	74.60 (16)	O3—S—C11—C16	28.16 (19)
S—O3—C1—C2	−177.32 (13)	O2—S—C11—C12	−35.86 (19)
C4—O4—C2—C1	−122.29 (19)	O1—S—C11—C12	95.84 (18)
C4—O4—C2—C3	−2.1 (2)	O3—S—C11—C12	−153.41 (16)
O3—C1—C2—O4	−64.2 (2)	C16—C11—C12—C13	0.1 (3)
O3—C1—C2—C3	−179.29 (16)	S—C11—C12—C13	−178.31 (16)
C4—O5—C3—C2	32.7 (2)	C11—C12—C13—C14	−1.5 (3)
O4—C2—C3—O5	−18.5 (2)	C12—C13—C14—C15	1.6 (3)
C1—C2—C3—O5	99.1 (2)	C12—C13—C14—C17	−177.4 (2)
C3—O5—C4—O4	−34.7 (2)	C13—C14—C15—C16	−0.3 (3)
C3—O5—C4—C6	−151.42 (19)	C17—C14—C15—C16	178.8 (2)
C3—O5—C4—C5	83.0 (2)	C14—C15—C16—C11	−1.0 (3)
C2—O4—C4—O5	22.3 (2)	C12—C11—C16—C15	1.1 (3)
C2—O4—C4—C6	138.77 (18)	S—C11—C16—C15	179.51 (16)
C2—O4—C4—C5	−97.16 (19)		

### Hydrogen-bond geometry (Å, °)

**Table:**

*D*—H···*A*	*D*—H	H···*A*	*D*···*A*	*D*—H···*A*
C16—H16···O1^i^	0.95	2.60	3.212 (2)	122
C17—H17C···O1^ii^	0.98	2.65	3.369 (3)	131
C17—H17B···O1^iii^	0.98	2.66	3.558 (3)	153
C5—H5A···O2^iv^	0.98	2.64	3.509 (3)	148

Symmetry codes: (i) *x*, *y*−1, *z*; (ii) −*x*, *y*−1/2, −*z*+1/2; (iii) *x*, −*y*+3/2, *z*−1/2; (iv) *x*, −*y*+3/2, *z*+1/2.
